# Functional Microendoscopy Reveals Calcium Responses of Single Cells in Tracheal Tuft Cells and Kidney Podocytes

**DOI:** 10.1002/smll.202411341

**Published:** 2025-04-01

**Authors:** Tobias A. Dancker, Mohamed Ibrahem Elhawy, Ramona Rittershauß, Qinghai Tian, Yvonne Schwarz, Markus D. A. Hoffmann, Christopher Carlein, Amanda Wyatt, Vanessa Wahl, Daniel Speyerer, Alaa Kandah, Ulrich Boehm, Leticia Prates Roma, Dieter Bruns, Peter Lipp, Gabriela Krasteva‐Christ, Marcel A. Lauterbach

**Affiliations:** ^1^ Molecular Imaging Center for Integrative Physiology and Molecular Medicine (CIPMM) Saarland University Kirrberger Str. 100, building 48 66421 Homburg Saarland Germany; ^2^ Institute of Anatomy and Cell Biology Saarland University Kirrberger Str. 100, building 61 66421 Homburg Saarland Germany; ^3^ Molecular Cell Biology, Center for Molecular Signaling (PZMS) Saarland University Kirrberger Str. 100, building 61 66421 Homburg Saarland Germany; ^4^ Molecular Neurophysiology, Center for Integrative Physiology and Molecular Medicine (CIPMM) Saarland University Kirrberger Str. 100, building 48 66421 Homburg Saarland Germany; ^5^ Biophysics, Center for Human and Molecular Biology (ZHMB) Saarland University Kirrberger Str. 100, building 48 66421 Homburg Saarland Germany; ^6^ Experimental Pharmacology, Center for Molecular Signaling (PZMS) Saarland University Kirrberger Str. 100, building 45&46 66421 Homburg Saarland Germany; ^7^ Center for Gender Specific Biology and Medicine (CGBM) Saarland University Kirrberger Str. 100 66421 Homburg Saarland Germany

**Keywords:** functional fluorescence microendoscopy, GRIN lens, image processing, intravital microscopy, kidney podocyte, tracheal brush cells, tracheal tuft cells

## Abstract

Microendoscopy, a crucial technology for minimally invasive investigations of organs, facilitates studies within confined cavities. However, conventional microendoscopy is often limited by probe size and the constraint of using a single excitation wavelength. In response to these constraints, a multichannel microendoscope with a slender profile of only 360 µm is engineered. Functional signals both in situ and in vivo are successfully captured from individual single cells, employing a specially developed software suite for image processing, and exhibiting an effective resolution of 4.6 µm, allowing for the resolution of subcellular neuronal structures. This system enabled the first examination of calcium dynamics in vivo in murine tracheal tuft cells (formerly named brush cells) and in situ in kidney podocytes. Additionally, it recorded ratiometric redox reactions in various biological settings, including intact explanted organs and pancreatic islet cultures. The flexibility and streamlined operation of the microendoscopic technique open new avenues for conducting in vivo research, allowing for studies of tissue and organ function at cellular resolution.

## Graphical abstract

A cost‐effective multicore‐fiber microendoscope for biological functional imaging is presented. The instrument is thin enough to image within the mouse trachea. It is applied to record responses of tracheal tuft cells to stimulation in vivo, as well as calcium and redox signals from various organs in situ. Additionally, the system's optical characterization, data processing pipeline, and multichannel capabilities are demonstrated.

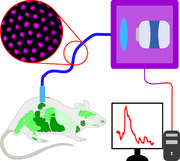

## 1 Introduction

Endoscopy is an important technique for minimally invasive imaging in both human and animal subjects, often facilitating concurrent biopsy collections.^[^ ^1^, ^2^ ^]^ Enhanced with appropriate light sources and filters, an endoscope becomes a powerful tool for analyzing tissue fluorescence. This capability contributes to tumor stratification, whether using the autofluorescence spectra of tumors or specific fluorescent dyes.^[^ ^1^, ^3^ ^]^ The ready availability of genetically modified mouse lines expressing fluorescent proteins, along with the vast potential for gene manipulation in these models, necessitates endoscopes compatible with mouse research. However, conventional endoscopes face limitations in preclinical rodent studies because of probe size, leading to a shift toward microendoscopes. Although recent microendoscopes designed for mouse models have been introduced, they often lack multiple excitation capabilities and sufficient flexibility and are rarely applied in vivo.^[^ ^4^, ^5^, ^6^, ^7^, ^8^, ^9^, ^10^, ^11^, ^12^ ^]^ Recent models overcome some of these limitations but remain often limited by their size, with fiber diameters exceeding 500 µm, making them unsuitable for small organs or the narrow lumina of mice.^[^ ^13^, ^14^, ^15^, ^16^, ^17^, ^18^ ^]^ Single multimode fiber systems represent a notable application of optical technology,^[^ ^19^, ^20^ ^]^ achieving probe diameters as small as 105 µm. However, these systems require highly stable, narrow‐band lasers and extensive calibration, which must be adjusted for each bending state of the probe. The endoscopes must therefore either remain rigid or readjust the image transmission for each bending change, leading to endoscopes that are either short (<3 cm) and stiff or require adjustments on a second to minute timescale after each bending change.^[^ ^20^ ^]^ A small microendoscope featuring multiple channels and equipped with a multicore fiber would provide a literally more flexible solution and represent a significant advancement. Such a system would enable minimally invasive in vivo tissue or organ examinations outside the skull and support longitudinal studies with reduced sample sizes. Such studies might encompass cellular or molecular responses to pharmaceuticals, cell metabolism, or reactions to environmental stimuli.^[^ ^10^ ^]^

Here, we concentrate particularly on two cell types reacting to such stimuli: podocytes and tuft cells. Additionally, we investigate whole explanted tissues and tissue/cell cultures in multi‐channel and ratiometric recordings.

Podocytes are integral components of the glomeruli and play a crucial role in the primary blood filtration process within the kidney. Via their foot processes, they create filtration slits that maintain selective permeability, preventing cellular components from escaping the bloodstream.^[^ ^21^ ^]^ When podocytes age or undergo injury, they experience a loss in density and structural integrity, resulting in increased leakage from the blood into the proto‐urine.^[^ ^22^, ^23^ ^]^ The regulation of podocyte foot process number, density, structure, and cytoskeleton is associated with calcium signaling cascades, which are modulated by angiotensin II stimulation. Injured podocytes exhibit increased sensitivity to these signals.^[^ ^24^, ^25^, ^26^ ^]^

Tracheal tuft cells – formerly known as brush cells – have been identified as chemosensory entities in recent studies^[^ ^27^, ^28^, ^29^ ^]^ and are characterized by their tuft of microvilli extending into the tracheal lumen. These cells are involved in detecting bitter substances in the murine trachea (500–1000 µm in diameter), influencing respiratory rates via cholinergic transmission^[^ ^30^ ^]^ and acting as paracrine messengers within autocrine signaling frameworks.^[^ ^31^ ^]^ Their activation is also linked to the triggering of local innate immune responses, particularly the mobilization of neutrophils in response to the stimulation of nociceptor sensory neurons, positioning them as a critical line of defense against infections.^[^ ^32^ ^]^ Denatonium benzoate is a tuft cell agonist^[^ ^32^ ^]^ that initiates a calcium signaling cascade through the TRPM5 channels (transient receptor potential cation channel subfamily M member 5 channels). The reaction to this bitter substance underscores the physiological importance of these cells, but its agonistic action has not been directly observed in vivo.

The reduction/oxidation‐sensitive green fluorescent protein 2 (roGFP2) is a ratiometric fluorophore derived from jellyfish green fluorescent protein (GFP), engineered to alter its excitation properties based on the redox state of the molecule.^[^ ^33^ ^]^ This characteristic renders roGFP2 valuable for the ratiometric investigation of redox dynamics across various cellular models.^[^ ^34^, ^35^ ^]^ Via genetic targeting, roGFP2 can be localized to specific cellular compartments, including the cytosol, mitochondrial intermembrane space, and the mitochondrial matrix (mito‐roGFP2).^[^ ^36^ ^]^ When fused with the peroxidase‐1 protein (Orp1), the resulting mito‐roGFP2‐Orp1 biosensor effectively detects H_2_O_2_‐specific fluorescence changes,^[^ ^37^ ^]^ providing a powerful means to explore redox variations in both isolated structures and whole tissues.

In this study, we present a microendoscope that shifts perspective by focusing on achieving cellular imaging in vivo without the skull for anchoring. Featuring multiple color channels and a small probe diameter of only 360 µm, this microendoscope has enabled us to successfully record functional calcium signals in tuft cells of the intact trachea in situ and in vivo, even in the presence of breathing related movement. To our knowledge, this represents the first publication of in vivo signals from these cells. Additionally, we recorded similar signals in kidney podocytes in situ. Our application also extended to recording mitochondria‐associated ratiometric redox signals in excised pancreas and kidney as well as in cultured pancreatic islets. Finally, the microendoscope's unique frame‐interleaved dual‐channel imaging capability enabled functional recordings of neurons expressing a green calcium indicator and a static red fluorescent protein, demonstrating its effectiveness in enhancing imaging fidelity with the use of a reference channel.

## 2 Results

### 2.1 Microendoscope Hardware and Software

**Figure 1:**
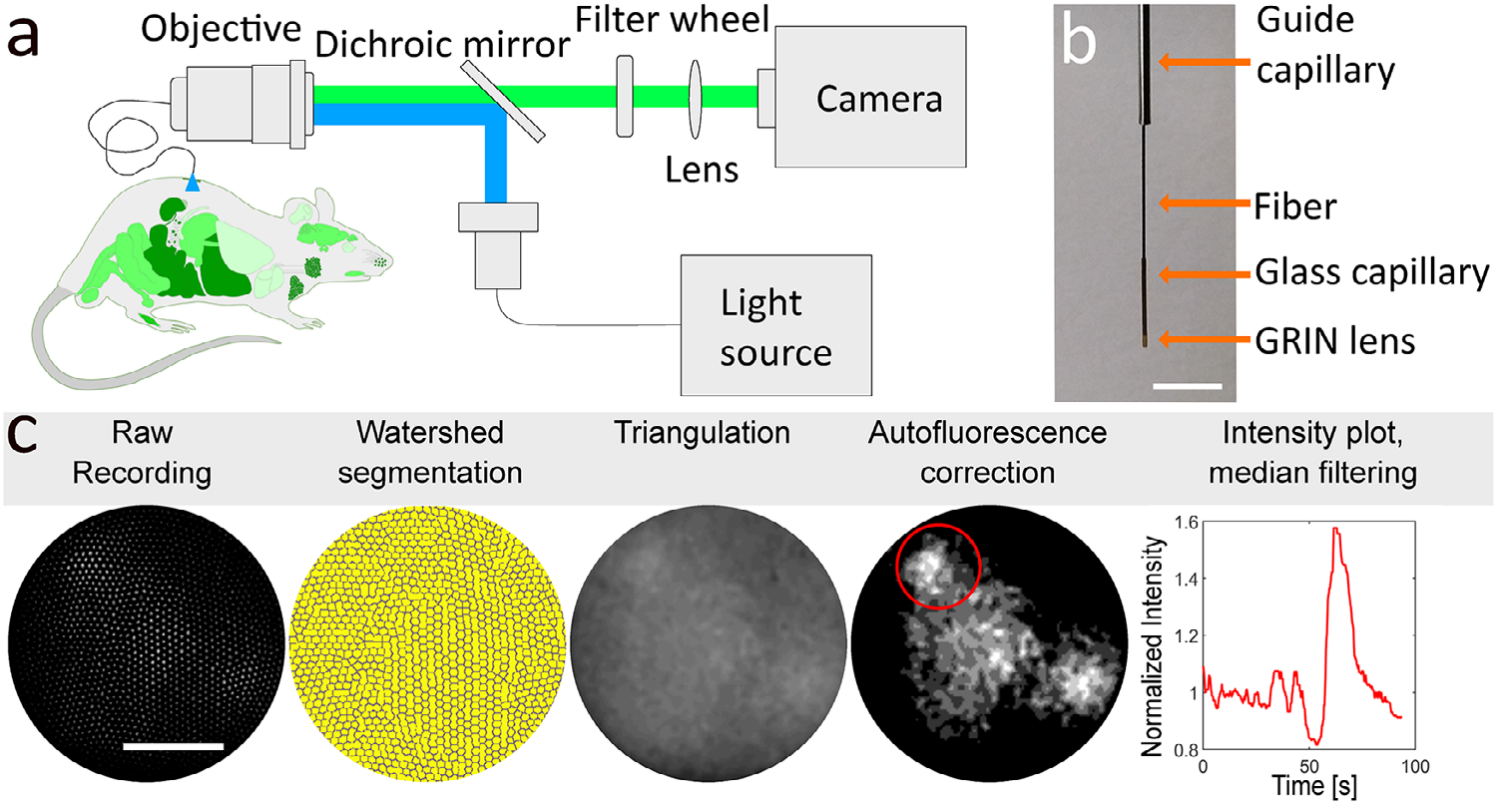
Microendoscope setup and image processing workflow. a) Schematic of the microendoscope system. b) Detailed view of the fiber end, highlighting the GRIN lens at the fiber's terminus, the glass capillary securing it, and the guide cannula. Scale bar: 5 mm. c) Initial raw images obtained through the multicore fiber are segmented to delineate each core's periphery. Pixel intensities within each core are averaged, and a smooth image is produced using triangulation‐based linear interpolation. The 25th intensity percentile is then subtracted from each frame to mitigate fiber autofluorescence. Subsequently, a ROI‐based intensity profile is plotted along the time dimension and smoothed with a sliding window median filter. Scale bar: 50 µm.

We designed our microendoscope (**Figure** **1**a,b) to balance minimizing probe diameter, achieving 360 µm, while maintaining an adequate field of view (FOV, 145 µm) and working distance (110 µm) for cellular imaging within targeted organs. This was achieved using a single gradient index (GRIN) lens fused to the end of a multicore fiber with 1460 cores (FIGH016‐160S, Fujikura, Surrey, United Kingdom, NA 0.4) (Figure 1b, see Experimental section for details). A three‐axis micromanipulator was employed for precise positioning of the fiber's imaging end over the sample. The probe was secured in a guide tube (Figure 1b). To facilitate position adjustments during animal experiments, a tilting sample stage allowed rotation along the horizontal and vertical axes and coarse height adjustments. The opposite end of the fiber bundle was projected onto a CCD camera in a 4f configuration (Figure 1a).

To select an appropriate dichroic mirror and filters for the Fujikura imaging fiber bundle, we referred to the work of Udovich et al., who measured the autofluorescence spectrum under 488 nm excitation.^[^ ^38^ ^]^ Our selection included a fixed multiband dichroic mirror and two alternating emission filters. We used a quad‐band emission filter for multi‐emission imaging and a single‐band filter to exclude the red autofluorescence peak during the imaging of GFP‐derived fluorophores (Figure S1, Supporting Information).

Illumination was provided by a Lightengine Aura III light source (Lumencor, Beaverton, OR, USA), which contains five individual illuminants, each with its own fixed excitation filter: 395/25, 475/28, 555/28, 635/22, and 730/40 nm (in the experiments presented here, only the first three channels were used). These channels are already combined in the light source and are always coupled to the light output path; a combined beam is delivered via a light guide with 3 mm diameter. The individual channels can be switched on and off independently without moving parts and therefore with sub‐millisecond time resolution via TTL (transistor–transistor logic) signals. Images were recorded by a monochrome 16‐bit CCD camera, ensuring sensitive detection of fluorescent signals.

The electronic design of our microendoscope incorporated electronic frame‐interleaved excitation switching. This enabled alternation between different excitation wavelengths on a frame‐by‐frame basis, while the emission of all channels passed through the same multiband filter. This approach allowed for the frame‐interleaved measurement of multiple color channels without complications from spatial shifts between channels or the need for slow filter switches.

### 2.2 Image Processing

The image analysis pipeline was designed to enhance the quality of our recordings (Figure 1c). It was responsible for eliminating visible individual fiber cores within the image and removing fiber autofluorescence.

To obtain clear images devoid of the honeycomb pattern of individual fiber cores, a custom MATLAB routine was developed. This routine begins with segmentation of the individual cores (see Experimental section). The fluorescence intensity within each identified core region is averaged, and triangulation‐based linear interpolation is used to generate interpolated images reduced to 128 × 128 pixels. This downsampling minimizes unnecessary oversampling of the fiber bundle's individual cores, consequently reducing data storage and processing requirements.

To further enhance image quality by improving contrast and reducing noise, the 25th intensity percentile was subtracted from each frame (see Experimental section). This approach allowed the brightness of each image to be adjusted individually, thereby reducing the effects of camera flicker artifacts. Given the prominence of autofluorescence, instability in recorded fluorescence intensity when multiplied by the substantial autofluorescence, could potentially introduce flickering artifacts into the time course of signal intensity. To address this issue, a per‐frame proportional subtraction was implemented rather than using a single baseline value.

To mitigate motion artifacts in in vivo experiments, cross‐correlation based image registration was applied (see Experimental section for details).

For the evaluation of functional fluorescence signals from images processed and corrected for autofluorescence, regions of interest (ROIs) specific to cells were delineated manually. Within these ROIs, intensity profiles were calculated and denoised using a moving window median filter.

### 2.3 Optical Characterization of the Microendoscope

**Figure 2:**
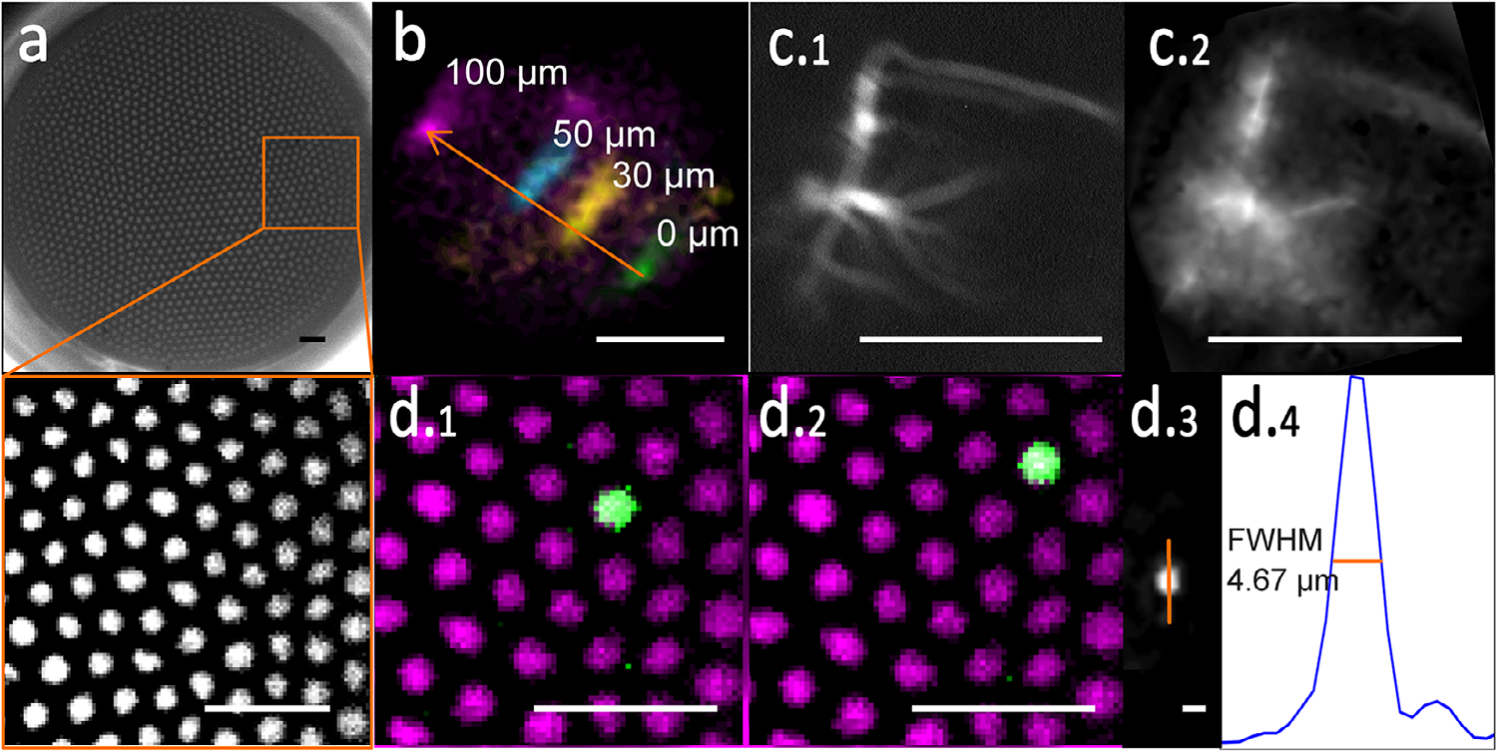
Optical characterization of the microendoscope. a) Microendoscope fiber bundle, with single fiber cores measuring 2.34 µm in diameter. Scale bars: 10 µm. b) Magnification and FOV calibration using a test sample (fluorescent fibers) observed before (green) and after shifts of 100 µm (purple), 50 µm (cyan), and 30 µm (yellow) via the micromanipulator. The shift on the camera was used to calculate the magnification and FOV. Scale bar: 50 µm. c) Validation of single‐cell resolution through simultaneous imaging of the same neuron with a conventional microscope (c.1) and the microendoscope (c.2). Scale bars: 100 µm. d) Resolution measurement with microspheres: d.1) In an unprocessed image, a 1 µm bead (green) appears on only one of all fibers (magenta, intensity‐scaled reference image without sample). d.2) After shifting by one core's distance, the bead appears on the adjacent fiber. d.3) Processed image with the honeycomb pattern removed. d.4) Intensity profile across the bead marked in d.3. Scale bars for d.1 and d.2: 10 µm; for d.3: 5 µm.

In the first series of experiments, we characterized the optical performance of the endoscope. The system's theoretical magnification, based on the objective (f  =  3.6 mm) and tube lens (f  =  200 mm) specifications, along with the nominal magnification of 1 of the GRIN lens, was anticipated to be 55.56. The magnification measured by moving a fluorescent sample by a known distance (**Figure** **2**a,b) was 55.44 ± 0.53 at the sample‐to‐camera level, with pixel dimensions of 0.2883 ± 0.0029 µm per side within the sample plane.

The FOV diameter was determined to be 147.5 ± 1.7 µm, deviating only minimally from the manufacturer's specification (145 µm, translating to a total area of 16 500 µm^2^). By counting the cores, visible as distinct bright spots under uniform illumination, we identified 1460 cores, consistent with the lower end of the specification (1600 ± 10%), equating to one core per 11.3 µm^2^. Assuming an ideal hexagonal lattice arrangement, the expected center‐to‐center distance between cores would be 3.68 µm. Our experimental measurements revealed an average distance of 3.71 ± 0.08 µm (Figure 2a), only slightly deviating from theoretical predictions, and a core diameter of 2.34 ± 0.06 µm.

To assess the microendoscope's ability to resolve single cells, we simultaneously imaged the same neuron using both the microendoscope and a conventional inverted epifluorescence microscope for comparison. This comparison confirmed that the microendoscope could discern not only the neuronal soma but also some extending neurites (Figure 2c), highlighting its potential for detailed cellular observations.

Theoretically, the optical resolution of our microendoscope at a wavelength of 475 nm, given the fiber's limiting numerical aperture of 0.4, should be ≈720 nm. However, the actual spacing between fiber cores of 3.71 ± 0.08 µm (Figure 2a) limits the system's effective resolution, which is thus governed by the sampling density of the individual fibers.

For experimental characterization of the microendoscope's imaging performance, we used 1 µm fluorescent beads, which occupied single fiber cores (Figure 2(d.1)). By shifting the fiber by the distance of one core, the bead was observed to transition to an adjacent core (Figure 2(d.2)). After image processing to remove the visibility of individual cores, the beads exhibited a full width at half maximum (FWHM) of 4.67 µm (Figure 2(d.3),(d.4)). The computed average FWHM across five beads was 4.64 ± 0.05 µm, defining the effective resolution of our microendoscope and underscoring its proficiency in cellular imaging applications.

### 2.4 Imaging Calcium Signals of Podocytes in Intact Kidneys In Situ

**Figure 3:**
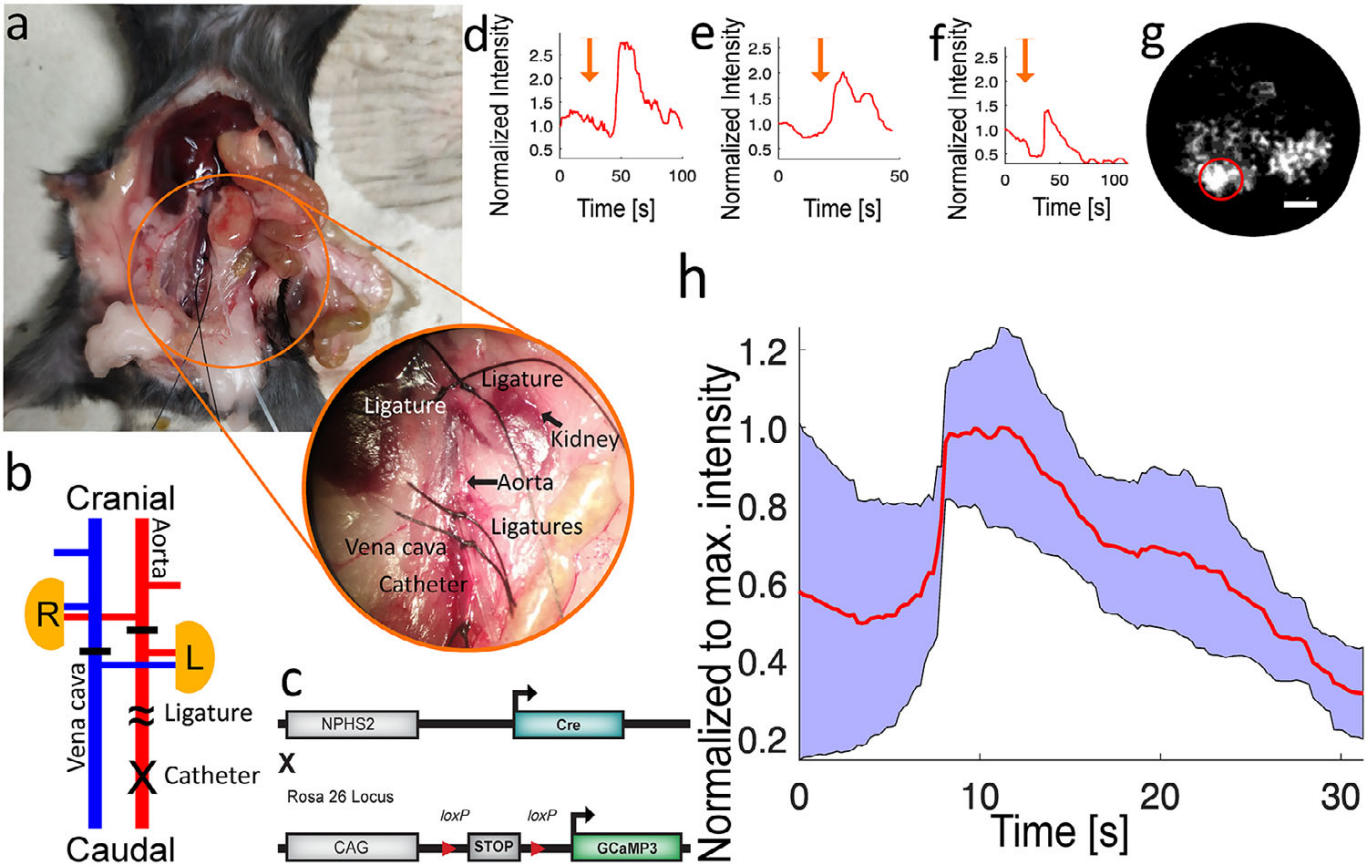
Functional calcium signals in mouse kidneys. a) Overview of the surgical procedure. b) Diagram illustrating the surgical approach designed to directly infuse a stimulation solution into the kidney, minimizing signal latency. The catheter (marked with X) is inserted into the infrarenal aorta, ensuring exclusive flow through the kidney by ligating all alternate vessels (indicated by –). The catheter is secured in place with two ligatures (∼). After positioning the microendoscope over the kidney and identifying podocytes by their basal GCaMP3 fluorescence, a 250 µL dose of 10 µM angiotensin II is administered. c) Genetic allele diagram. d–f) Varied calcium responses to angiotensin II stimulation, showing either an initial surge in signal intensity followed by a plateau phase before a decline (d, e) or a direct decrease in calcium levels (f). g) Processed microendoscope image corresponding to trace f. Scale bar: 20 µm. h) Average signals (red) from three separate stimulations, each from a different animal, accompanied by the standard deviation (blue shaded area). All data were captured at a rate of 4.3 frames per second (fps).

Following the optimization and comprehensive characterization of our microendoscope, we recorded functional signals within organs. Initially, we focused on capturing the calcium response in green fluorescent protein, calmodulin, and M13 (GCaMP3)‐expressing podocytes within intact kidneys. To stimulate these cells hormonally with angiotensin II, we inserted an arterial catheter into the infrarenal aorta of freshly euthanized mice. By occluding the superior mesenteric aorta, we ensured that injections through the catheter were directed exclusively to the kidneys, achieving targeted stimulation of the podocytes (**Figure** **3**a–c).

In this series, we stimulated three kidneys and recorded single‐cell calcium signals (Figure 3d–f). A representative experiment showcasing podocytes imaged with the microendoscope is presented in Figure 3g. The stimulation triggered notable increases in GCaMP3 fluorescence within individual podocytes, with variable time courses. We observed up to a twofold elevation in cell‐specific fluorescence intensity upon stimulation. Typically, this increase was preceded by a brief decline in signal intensity (<10 s), followed by a rapid rise within 1–3 s. Some responses plateaued briefly (<10 s) before a gradual reduction over the subsequent 15–20 s (Figure 3d,e), whereas others did not exhibit such a plateau (Figure 3f). The consistent signal rise across experiments is demonstrated by the plotted averages and their standard deviations (of one cell per animal) (Figure 3h).

### 2.5 Imaging Calcium Signals of Tracheal Tuft Cells In Situ

**Figure 4:**
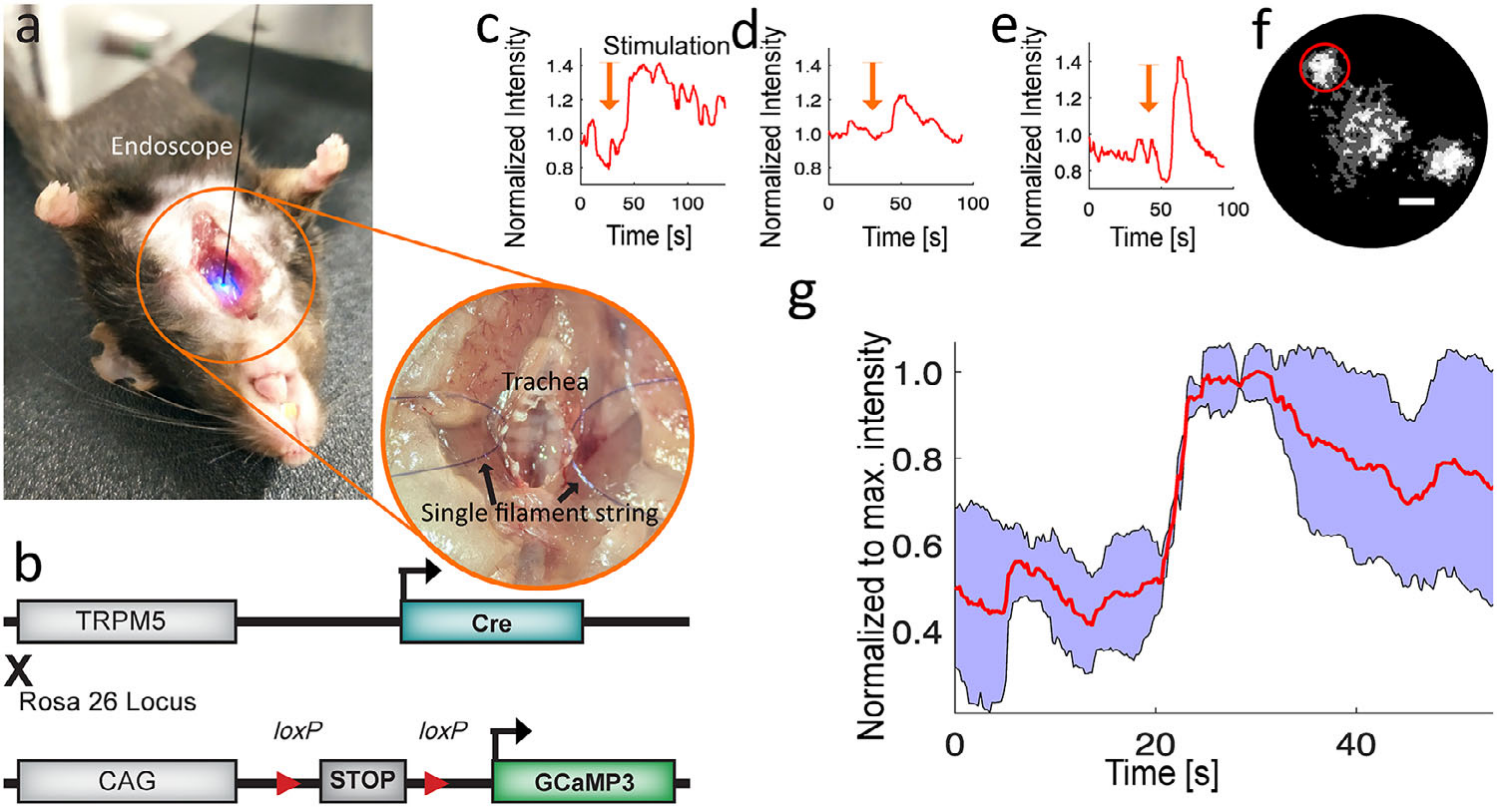
Functional calcium signals in mouse trachea. a) Insertion of the microendoscope probe into the trachea. b) Genetic allele diagram. c–e) Individual calcium responses to denatonium stimulation, characterized by a rapid increase in signal, followed by either a prolonged plateau (c) or an immediate decrease (d,e). f) Processed image from the microendoscope video corresponding to trace e. Scale bar: 20 µm (see also Video S1, Supporting Information). g) Average signal (red) from three stimulations, each from a different animal, with the standard deviation (blue shaded area). Data were recorded at a frame rate of 4.3 fps.

To demonstrate the microendoscope system's capability for in situ imaging within narrow cavities inaccessible to larger instruments, we recorded functional calcium signals from GCaMP3‐expressing tuft cells in the mouse trachea. After opening the skin, the salivary glands and sternothyroid muscles were positioned laterally, and the trachea was opened ventrally with a small incision. To increase the accessible tracheal surface area, a single filament string was anchored to a cartilage ring on each side of the trachea, effectively widening the opening (**Figure** **4**a). By tilting the sample/animal stage approximately 15° around the anterior‐posterior axis, we facilitated access to the tracheal wall side, which was anticipated to contain a higher concentration of tuft cells.^[^ ^32^ ^]^ Stimulation of the trachea was achieved by locally applying 4‐µL droplets of 10 mM denatonium benzoate. This led to a time‐dependent increase in intracellular calcium concentration within individual tuft cells (Figure 4b), as evidenced by the rising GCaMP3 signal (Figure 4c–e). Peak responses indicated approximately a 1.6‐fold increase in cell‐specific intensity. The signal intensity typically rose within the initial 1–3 s, occasionally plateauing before diminishing over the subsequent 20–50 s. The signal averages (of one cell per animal) revealed reproducible results (Figure 4g). The average of all frames showed the locations of individual tuft cells (Figure 4f), with the low initial intensity, stimulation‐induced intensity rise, and subsequent decline in fluorescence conspicuously observable in the corresponding video (Video S1, Supporting Information). To substantiate that the dynamic calcium signals originated from single cells, we imaged the distribution of tuft cells within a fixed, whole‐mount trachea, which confirmed the sparse distribution of individual tuft cells throughout the trachea (Figure S2, Supporting Information).

### 2.6 Imaging Calcium Signals of Tracheal Tuft Cells In Vivo

**Figure 5:**
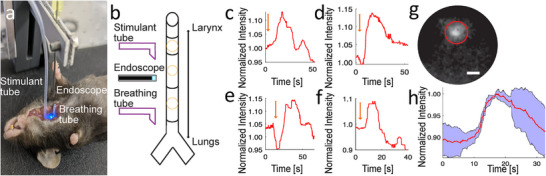
Functional calcium signals in the mouse trachea in vivo. a) Positioning of the stimulant tube (left) into the mouse trachea, with views of the microendoscope (center, emitting light) and the breathing tube (right, adjacent to the microendoscope probe). The stimulant tube is connected to a syringe pump. b) Schematic view of the in vivo surgery. Three small holes are cut into the trachea. Into the one proximal to the larynx the stimulant tube is inserted, into the one proximal to the lungs a breathing tube is inserted. Through the center hole the microendoscope is inserted. c–f) Individual in vivo calcium responses to denatonium stimulation (orange arrows), characterized by a rapid increase in signal intensity. g) Field of view with region of interest (ROI) corresponding to c (see also Video S2, Supporting Information). Scale bar: 20 µm. h) Average signal (red) from four stimulations, with standard deviation (blue shaded area). Data was recorded at a frame rate of 7.6 fps. The traces in panels c–f originate from four different cells across two animals, with c and d from one animal and e and f from the other.

We recorded GCaMP3 signals from individual tuft cells in vivo to assess the effect of denatonium benzoate on tuft cell calcium signaling in the living animal and to confirm the effectiveness of our microendoscope for in vivo imaging. After anesthetizing the mouse with urethane, we performed the surgery as described under in situ experiments. However, we decoupled a segment of the trachea from the animal's breathing by fitting it with a breathing tube. For on‐demand stimulation, a syringe pump connected to the cranial trachea segment via a tube was used. The microendoscope probe was inserted through a small hole (≈400 µm ⌀) in the intact trachea between the two sites (**Figure** **5**a,b). After identifying cells with the microendoscope, the trachea was stimulated with a 10 mM denatonium solution at a rate of ≈2 µL s^−1^. The tuft cells responded with a calcium signal showing up to a 15% increase in fluorescence intensity over a period of 3–10 s (Figure 5c–f, Video S2 (Supporting Information), whose average is shown in Figure 5g). Averaging these signals (two cells per animal) revealed a reproducible rise and subsequent fall in fluorescence intensity of ≈10% (Figure 5h). To our knowledge, these experiments represent the first characterization of the tuft cell response to denatonium benzoate in vivo.

### 2.7 Dual‐Channel Recordings

**Figure 6:**
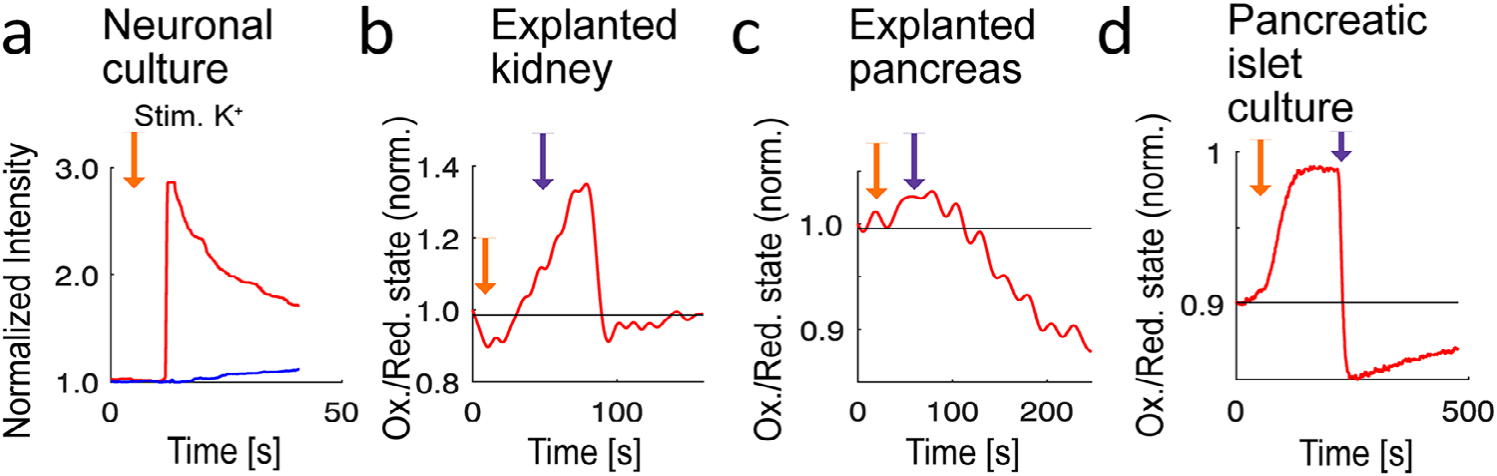
Multichannel recordings. a) Calcium response (red) of an individual neuron to 80 mM KCl, compared to the constant frame‐interleaved reference signal (blue), demonstrating expected stability. b) Ratiometric recording depicting redox state fluctuations within the excised kidney following sequential applications of an oxidizing agent (diamide, final concentration: 2 mM) and a reducing agent (dithiothreitol, final concentration: 10 mM). c) Ratiometric analysis of roGFP2‐Orp1 redox state changes in the excised pancreas, induced by the application of oxidizing (diamide, final concentration: 2 mM) and reducing (dithiothreitol, final concentration: 10 mM) agents. d) Faster and more pronounced roGFP2‐Orp1 redox state transitions within the islet, attributed to quicker diffusion. For b–d, the orange arrow indicates stimulation with diamide, and the purple arrow indicates stimulation with dithiothreitol.

Our investigations also assessed the frame‐interleaved dual‐channel recording capabilities of the microendoscope. First, we focused on neuronal cultures, genetically modified to co‐express the calcium indicator GCaMP6f and the static fluorophore mKate2. Upon inducing membrane depolarization with an 80 mM KCl solution, the GCaMP6f channel exhibited a 2.5‐fold increase in cell‐specific fluorescence intensity, indicating Ca^2^⁺ influx through voltage‐gated calcium channels. In contrast, the mKate2 reference fluorescence showed minimal variation (**Figure** **6**a), effectively ruling out motion artifacts as a source of fluorescence changes. These neuronal cultures were also used to prove that our microendoscope does not depend on genetically encoded calcium sensors, but can record calcium signals with chemical sensors, shown with Fluo‐4 AM signals (Figure S3, Supporting Information).

Our final set of experiments demonstrated the microendoscope's proficiency to detect ratiometric signals using roGFP2‐Orp1. The roGFP2‐Orp1 molecule, a GFP derivative, is excitable at two distinct wavelength ranges; its excitation efficiency depends on its oxidation state, primarily influenced by the presence of H_2_O_2_. Under oxidative conditions, roGFP2‐Orp1 shows high excitation efficiency at shorter wavelengths (≈400 nm), whereas reducing conditions result in higher excitation efficiency at longer wavelengths (≈490 nm).^[^ ^33^ ^]^

For ratiometric analyses within organ tissues, we used pancreas and kidney from mice ubiquitously expressing the mito‐roGFP2‐Orp1 sensor. Excised organs were first rinsed in Krebs‐Henseleit buffer (KHB) to remove any residual blood that might cling to the lens and cause autofluorescence.^[^ ^36^ ^]^ All specimens were then imaged in KHB using frame‐interleaved excitation at 395 nm and 475 nm wavelengths. The recorded movie was divided into two distinct time series based on the excitation wavelength. From these series, ROI‐based intensity traces were generated, and their ratios were calculated at each time point. The final trace was normalized to the mean of the initial 50 values. Oxidation was induced with diamide at a final concentration of 2 mM, and reduction was achieved using dithiothreitol (DTT) at a final concentration of 10 mM. These ratiometric measurements within the pancreas and kidneys showed the expected fluorescence signals of roGFP2‐Orp1 (Figure 6b,c). The organ recordings exhibited a delayed response, attributed to the diffusion time required for the reagents to permeate the tissue. In contrast, isolated pancreatic islet experiments demonstrated rapid reactions with short delays (Figure 6d), consistent with expectations because of the faster diffusion dynamics within the culture setup.

## 3 Summary and Discussion

Following the comprehensive characterization and validation of our particularly small microendoscope, we demonstrated several biological applications in in‐vivo and in‐situ experiments, including that individual tuft cells in the mouse trachea respond in vivo to a bitter substance. The development and characterization of our microendoscope (Figures 1 and 2) highlighted its effectiveness in capturing functional cellular fluorescence signals in challenging anatomical locations, such as the mouse trachea (Figures 4, 5). The system was further refined with an appropriate data processing pipeline (Figure 1), asserting its applicability across various organ systems with different biosensors (Figures 3, 4, 5, 6). Utilizing its frame‐interleaved multichannel capabilities, we successfully performed ratiometric and reference channel imaging in intact organs and cultures of neurons and pancreatic islets, showcasing the system's versatility (Figure 6). The microendoscope's ability to facilitate flexible, cellular‐resolution functional imaging in situ and in vivo represents a significant advancement over traditional methods that require organ explantation or lack single‐cell resolution.^[^ ^24^, ^39^, ^40^, ^41^, ^42^, ^43^ ^]^

Our system recorded fluorescence signals with shapes that closely matched those captured by higher‐resolution microscopes,^[^ ^28^ ^]^ demonstrating in vivo the stimulating effect of denatonium benzoate, previously observed only in explanted trachea.^[^ ^28^, ^32^ ^]^ This success in in vivo mouse endoscopy underscores the advantage of a small, flexible microendoscope for imaging cells without the need for anchoring to the skull or other bones.

Furthermore, our investigations in renal podocytes (Figure 3) highlighted the microendoscope's ability to detect and document renal calcium signals at the single‐cell level consistent in shape and time course with intravital and cultured podocyte calcium signals.^[^ ^44^, ^45^ ^]^

Podocytes, located within the superficial layers of the renal cortex—typically between the 2nd and 5th cell layers^[^ ^46^ ^]^—are accessible to the microendoscope because of its 110 µm working distance. Although our wide‐field epifluorescence configuration with LED source lacks the optical‐sectioning capabilities of confocal or two‐photon microscopy, the microendoscope's flexibility compensates for this limitation. Traditional confocal and two‐photon microscopes require sample relocation to the instrument, which can restrict the scope of in vivo studies. Microendoscopes with confocal and two‐photon configurations can overcome the resolution, field‐of‐view or sectioning capabilities of our system, but they typically come with a trade‐off in size.^[^ ^5^, ^8^, ^47^, ^48^, ^49^ ^]^ The diameter of a microendoscope can be reduced beyond our system by instruments that use a single multimode fiber; however, these systems require complex instrumentation, extensive calibration, and rigid endoscopes or constant tracking of the shape/bending changes with according compensation.^[^ ^19^, ^20^ ^]^

Designed specifically for functional imaging in soft tissue environments where stable anchorage, such as the skull, is not available, our microendoscope compensates for movement artifacts in several ways. First, its multichannel capacity not only enabled ratiometric measurements, but also the use of reference channels to effectively distinguish functional signals from motion artifacts^[^ ^50^ ^]^ (Figure 6a). The ability to simultaneously image up to four channels, accommodating a broad spectrum of fluorophores, positions our system as a versatile tool for future in vivo studies. Second, our advanced software routines effectively compensate for motion artifacts in single‐channel recordings. Lastly, surgical techniques were optimized to minimize motion artifacts from breathing. Motion artifacts could be further reduced by triggering image acquisition to the animal's breathing or heartbeat. Additional trigger signals, e.g. from an automatic pump, could be used for logging time stamps of biological stimulation, whereas stimulants were applied in our experiments mostly manually.

Although larger systems anchored to rigid points allow for cellular microscopy with a broader FOV and enhanced resolution,^[^ ^51^, ^52^ ^]^ our microendoscope presents a promising, less invasive alternative for cellular‐level imaging. It aims to reduce animal burden and to provide access to imaging sites that are otherwise inaccessible for larger, less adaptable systems.

Finally, our microendoscope could be integrated with additional imaging methodologies to enable comprehensive, simultaneous multi‐organ in vivo studies in the future. We demonstrated here simultaneous imaging of a single cell using both a conventional microscope and our microendoscope for resolution comparison (Figure 2c). This approach can be expanded for multisite imaging within a single subject: Choosing different regions/organs instead of the comparative imaging of the same FOV presented here, one organ can be imaged with the microendoscope, while another organ is simultaneously observed with a conventional microscope or a second endoscope. Conceptually similar to other multisite systems^[^ ^53^ ^]^ our dual‐system setup offers the advantage of decoupling the two light paths and observation sites. This configuration could use a conventional optical system for detailed visualization and potential optogenetic modulation of one organ while leveraging the microendoscope's agility to monitor functional responses in another organ concurrently. This dual‐imaging strategy broadens the scope of biological research, particularly in studying integral organs such as the kidneys, trachea, and pancreas, by providing a dynamic perspective on inter‐organ interactions and systemic physiological responses.

## 4 Experimental Section

### Microendoscope Materials

The microendoscope was assembled on a 600 mm × 900 mm breadboard (Newport, Darmstadt, Germany) using standard optomechanical components from Thorlabs (Newton, NJ, United States). The imaging system incorporated an imaging fiber bundle (FIGH016‐160S, Fujikura, Surrey, United Kingdom), which features a nominal 145 µm image circle and 1600 ± 10% fiber cores with a numerical aperture (NA) of 0.4 and a coating diameter of 210 µm. This fiber bundle was coupled with a GRIN lens (NEM‐025‐06‐00‐520‐S, Grintech, Jena, Germany) with a diameter of 250 µm, offering a 110 µm working distance in water, an NA of 0.5, and a nominal magnification of ‐1. These components were bonded with optical adhesive and secured within a polyimide‐coated glass capillary, 360 µm in diameter and 7 mm in length by Grintech (Figure 1b). This capillary defines the thickest section of the probe. A syringe cannula with a 0.60 mm inner diameter, filed off at the sharp end, served as a guiding tube for the probe (Figure 1b).

The imaging system used an Evolve 512 monochrome 16‐bit CCD camera (Photometrics, Tucson, AZ, USA). The 4f configuration for imaging the fiber bundle onto the camera employed a 50× objective (MPLFLN50X, Olympus, Shinjuku City, Japan) with a 3.6 mm focal length and an NA of 0.8, complemented by a doublet lens with a 200 mm focal length (AC254‐200‐A‐ML, Thorlabs).

The system was equipped with a multiband dichroic mirror (F67 401, AHF analysentechnik, Tübingen‐Pfrondorf, Germany), a quad‐band emission filter (435/15, 520/10, 595/15, and 695/30; F67 401, AHF analysentechnik), and alternatively, a 511/20 single‐band filter (F39‐509, AHF analysentechnik). The emission filters, excitation wavelengths, and fluorophores used in the different experiments are listed in Table S1 (Supporting Information).

Illumination was provided by a Lightengine Aura III (Lumencor, Beaverton, OR, USA), which contains five individual light sources, each with its own fixed excitation filter (395/25, 475/28, 555/28, 635/22, 730/40). The combined output light is delivered via one light guide (3 mm diameter, 1.5 m length, L52‐4LG15, AHF analysentechnik) with a collimator (L55‐COL, AHF analysentechnik) on its distal end. All color channels can be switched with sub‐millisecond time resolution via electronic TTL signals sent to the Lightengine Aura III, because all internal light sources are always simultaneously coupled to the output light path and no filter switching is needed to change channels. An Arduino UNO (Arduino, Monza, Italy) was programmed in its native language to enable frame‐interleaved excitation. The camera's frame‐exposure TTL signal triggered the Arduino to allocate these TTL signals to distinct excitation LEDs, with each LED's activation frame configurable via a graphical user interface (GUI). Sequential excitations for frame‐wise switching could be configured, enhancing the system's flexibility and responsiveness to experimental needs. An automatic reset of the excitation sequence occurred if no TTL signal from the camera was detected for a 5‐s interval, ensuring consistent color channel assignments by the Arduino across recordings, with each session starting with a predetermined excitation wavelength.

For camera operation and image capture, Micromanager 2.0 software was employed, whereas image processing was performed using custom routines developed in MATLAB (MathWorks, Natick, MA, USA).

### Image Processing

Image processing began with identifying each fiber core's position using a reference image where the fiber cores were distinctly visible. Suitable reference images were obtained from 395 nm excitation, which induced strong autofluorescence, from images of a homogeneous sample, or from the average image of an extended time series. The reference image was then refined by convolving it with a 2D Gaussian function to reduce noise. Subsequently, the MATLAB watershed command was applied on the intensity‐inverted images for segmentation. This delineates the regions around the local intensity maxima of each fiber core and gives thus a segmentation of each individual core. These cores are roughly (but due to the manufacturing imperfections not perfectly) arranged in a hexagonal lattice. For further processing and display, images with square pixels in a Cartesian arrangement are needed. Therefore, the average fluorescence intensity of the segmented regions at the core positions was interpolated with triangulation‐based linear interpolation onto a 128 × 128 2D grid using MATLAB's griddata command for every frame in the time series.

To further improve image quality by reducing noise, the 25th percentile of intensity values was subtracted from each frame. This approach was chosen because subtracting the 25th percentile represented the image's dark regions while avoiding the significant noise vulnerability associated with using the minimum intensity value. Dark corners outside the circular fiber bundle were excluded from the percentile calculation.

To mitigate motion artifacts in in vivo experiments, cross‐correlation‐based image registration was performed using custom MATLAB routines if movement had occurred during the recording. The cross correlation of each movie frame relative to the first frame of the video was calculated: For each movie frame, its mean intensity was subtracted. Then the cross‐correlation with the first (reference) frame was calculated (MATLAB's command normxcorr2) and the position of the maximum in the cross correlation was determined. Finally, each frame was shifted by the difference of this maximum‐position from the center. To ensure that the cross correlation was dominated by features in the image rather than the border of the round fiber bundle, regions outside the fiber bundle were first replaced with the mean intensity within the round FOV.

For calculating ΔF/F intensity profiles, the spatial mean intensity within the ROI across the time series was normalized to the series' lowest intensity and then subtracted by one.

A moving window median filter with a window spanning 30 frames for in situ recordings and 53 frames for the faster in vivo recordings was applied for denoising. A side‐by‐side comparison of raw and median‐filtered data is provided in Figure S4 (Supporting Information).

Aligning single‐cell signals at their onset was crucial for accurately determining signal variance. The onset of the intensity surge was identified as the earliest moment when the intensity exceeded 80% of its maximum value (96% in the case of the in vivo recordings due to weaker signals), ensuring uniform alignment of all signals at this point for subsequent analyses.

Supplementary videos were exported as an .avi file via ImageJ with their original frame rates. They were then imported into Microsoft Clipchamp, cut, cropped, and equipped with a stimulation indicator. For export, they were interpolated to 30 frames per second (fps) for real time viewing.

### Optical Characterization

To count the fibers within the bundle (Figure 2a), an image was captured using 395/25‐ nm excitation and 511/20‐ nm emission, revealing the autofluorescence of the fibers with no sample present. The exposure time was set to 200 ms. After thresholding, the objects were counted using a custom MATLAB routine to determine the number of fiber cores.

To characterize the FOV and magnification of the microendoscope, a fluorescent text marker (Stabilo Boss Pink, Heroldsberg, Germany) was applied to optical cleaning tissue (MC‐5, Thorlabs) and allowed to dry. The fluorescent tissue fibers were then imaged using the microendoscope under 475/25 nm excitation. By precisely moving the sample using a micromanipulator, the sample was displaced by 30 µm, 50 µm, and 100 µm (Figure 2b), and the resultant pixel displacement of the fibers was determined using the Pythagorean theorem. The pixel size was calculated by dividing the micromanipulator's movement distance by the resultant pixel count. Magnification was determined by multiplying the pixel distance by 16 µm (the size of a pixel on the camera chip) and dividing by the micromanipulator's movement distance. Standard deviations from these measurements were also computed.

To evaluate the microendoscope's single‐cell resolution capabilities in comparison to those of a conventional microscope (Figure 2c), we simultaneously imaged live GFP‐positive neurons using both the microendoscope and an inverted microscope (IX 83, Olympus) in wide‐field epifluorescence mode. The inverted microscope was equipped with a 490 nm LED light source (CoolLED, Andover, United Kingdom) and a 405/488/561/635 BrightLineLaser quad‐band filter set (F66 866, AHF analysentechnik). The neurons were prepared on a cover slip and placed on the sample stage for imaging with a 40× objective (UPLXAPO40XO, Olympus, oil immersion, NA 1.4). After identifying a target cell with the inverted microscope, the microendoscope's fiber was precisely aligned over the same cell to capture images. Images were taken using the epifluorescence microscope with an exposure time of 219 ms and the microendoscope with an exposure time of 2.5 s. For optimal visualization in Figure 2c, the resultant images were averaged over five captures.

The microendoscope's resolution was further assessed by imaging 1 µm yellow‐green (505/515) FluoSpheres (Life Technologies Corporation, Eugene, OR, USA) suspended on poly‐D‐lysine‐coated microscopy slides (Figure 2d). A 100 µL droplet of 5 mg mL^−1^ poly‐D‐lysine solution (Serva Electrophoresis GmbH, Heidelberg, Germany) was applied to a Superfrost Plus adhesion microscope slide (Epredia, Essendonk, Netherlands) and allowed to sit for 10 min at room temperature. After washing the slide with tap water and allowing it to dry, a 10 µL droplet of FluoSpheres diluted 1:5000 in water was applied to the poly‐D‐lysine residue and left to incubate for another 10 min at room temperature before being rinsed. This preparation was left unmounted to ensure effective access to the imaging surface by the microendoscope.

### Spectra

Excitation spectra (Figure S1, Supporting Information) were captured using a fiber‐coupled spectrometer (CCS200, Thorlabs). Emission filter spectra were obtained directly from the vendor (AHF.de), and the eGFP spectrum was retrieved from fpbase.org.^[^ ^54^, ^55^ ^]^

### Animals

The mice used in our experiments were bred and housed in facilities certified by the Saarland State Office for Health and Consumer Protection. They were maintained on a 12 h light/dark cycle and had unlimited access to water and food. The colony was kept in an open environment, with mice housed in groups of two to five per cage, including both male and female specimens. All animal care and handling adhered to German guidelines for the welfare and use of laboratory animals. Euthanasia procedures, approved for organ collection under Section 4, paragraph 3 of the German Animal Welfare Act, were followed, with experiments conducted immediately post‐mortem. Anesthesia and subsequent in vivo experiments were performed in accordance with animal license 01/2023 from the aforementioned State Office.

### Podocyte Calcium Imaging

Functional single‐cell data were recorded from the kidneys of mice expressing the calcium sensor GCaMP3 in NPHS2‐positive cells, i.e., podocytes. This targeting was achieved by breeding an NPHS2‐Cre mouse^[^ ^56^ ^]^ (Jackson strain number 0 08205, The Jackson Laboratory, Bar Harbor, ME, USA) with a mouse carrying a transcriptionally silent GCaMP3 gene at the ROSA26 locus^[^ ^57^ ^]^ (Jackson strain number 02 8764, generously provided by Dr. Dwight Bergles, Johns Hopkins University School of Medicine, USA), resulting in exclusive GCaMP3 expression in renal podocytes (Figure 3c), with all podocytes being heterozygous for the GCaMP3 allele.

To provide direct access for podocyte stimulation within the kidney, the abdominal cavity was carefully opened following anesthesia with 4 % isoflurane and cervical dislocation. To direct pharmacological agents precisely to the kidneys, an arterial catheter (Intramedic PE10, BD Laboratories, Mississauga, Canada) was placed between the renal and inferior mesenteric arteries within the infrarenal aorta. This placement was complemented by ligating both the superior mesenteric aorta and inferior vena cava, cranial to the renal vein, and removing the renal capsule. This surgical technique, adapted from Czogalla et al.,^[^ ^58^ ^]^ was designed to ensure direct perfusion of the kidney, allowing the reagent to effectively reach the podocytes via the microvasculature. All surgical tools were sourced from Fine Science Tools (Heidelberg, Germany).

For imaging podocytes, extraneous fat and connective tissues were retracted from around the kidney. The microendoscope probe, held in place by the micromanipulator, was carefully positioned above the kidney. GCaMP3‐positive podocytes were identified by gradually lowering the probe toward the kidney surface until fluorescence signals were visible on the camera. The interval from animal euthanasia to the start of imaging was kept within 60–80 min, reflecting the typical timeframe of kidney transplantation procedures, thus suggesting preserved organ viability.^[^ ^59^ ^]^

Podocyte activation was achieved by perfusing the intact kidney via the arterial catheter with 250 µL of a 10 µM solution of human angiotensin II (Sigma‐Aldrich, Darmstadt, Germany, A9525), prepared in a specially formulated kidney buffer (10 mM NaHCO_3_, 15 mM KCl, 100 mM KH_2_PO_4_, 198 mM D‐glucose, pH adjusted to 7.0 with KOH). To remove residual autofluorescent blood and prevent cross‐stimulation effects, the kidney was flushed with 250 µL of the kidney buffer before the initial stimulation and between subsequent stimulations. The stimulant was manually injected through the catheter using a syringe, a process that introduced variability in the latency periods preceding cellular activation.

Imaging was performed at a frame rate of 4.3 fps, with each frame exposed for 200 ms, using the 475/28 nm excitation light source paired with a 511/20 nm emission filter. The intensity of the 475/28 nm LED at the imaging plane was 47 ± 3 µW.

### Tuft Cell Calcium Imaging

For the experiments in the mouse trachea, we recorded the responses of single TRPM5‐positive cells, i.e., tuft cells, to the bitter compound denatonium benzoate. Specific expression of the calcium sensor GCaMP3 in tuft cells was achieved by crossing a TRPM5‐Cre mouse^[^ ^60^ ^]^ with a Reverse Oriented Splice Acceptor (ROSA26)‐locus‐based, transcriptionally silenced GCaMP3‐gene carrier mouse^[^ ^57^ ^]^ (Jackson strain number 02 8764) (Figure 4b). The Cre‐mediated recombination process ensured the exclusive expression of GCaMP3 in cells expressing TRPM5, at least transiently, with all imaged tuft cells being heterozygous for the GCaMP3 allele.

Functional signals from tuft cells in the mouse trachea were imaged using the microendoscope following a minimally invasive preparation adapted for in situ and in vivo experiments. For in situ imaging, i.e., post‐mortem, the goal was to expose as much of the tracheal surface as possible, and breathing‐related movement artifacts were not a concern. The procedure began by anesthetizing the animal with 4 % isoflurane and was followed by cervical dislocation. Next, the anterior cervical skin was shaved and the head was gently stretched using a string tied behind the incisors. A small ventral incision was made to expose the salivary glands, which were then gently retracted. The sternothyroid muscles were pierced with Dumont forceps and retracted. A ventral cut was made along the length of the trachea. Single filament strings were secured around individual cartilage rings on either side of the cut to pull the trachea further open. These filaments were then taped down to provide a large accessible region by keeping the trachea continually open (Figure 4a). in situ experiments were conducted within 20 min post‐mortem to preserve tuft cell functionality.

To record functional tuft cell signals in vivo, it was necessary to minimize the impact of the animal's breathing on the imaged region of the trachea, requiring an adapted surgical procedure. To ensure minimal harm to the animal, the microendoscope's entry hole was kept as small as possible. The animals were anesthetized with urethane (1.5 g kg^−1^), which is preferable to ketamine‐xylazine in terms of maintaining natural respiration.^[^ ^61^, ^62^ ^]^ Once adequate anesthesia was confirmed by the absence of the toe pinch reflex, the animal's throat was shaved, and protective eye ointment was applied. The animal was then placed on a surgical heating mat. The surgical procedure involved opening the skin, retracting the salivary glands, and piercing and moving aside the sternothyroid muscles. A ventral hole, as large as needed to insert a tube (800/110/260, Portex/Fisher Scientific, Schwerte, Germany; outer diameter 1.27 mm, inner diameter 0.86 mm), was cut ventrally and as caudal as possible. A bent tube was inserted through this hole to allow the animal to breathe while isolating the trachea caudal to the tube from the animal's respiration. A second hole was cut cranially, near the larynx, into which a tube for stimulant injection was placed. These procedures enabled stimulation of the isolated trachea segment with a liquid stimulant. A third, small hole was made in the isolated trachea segment, ensuring it was small enough to avoid damage to surrounding nerve fibers (≈400 µm diameter, compared to the microendoscope's maximum diameter of 360 µm). Through this hole, the microendoscope probe was inserted (Figure 5a,b).

For the in situ (post‐mortem) experiments, tuft cells were exposed to 4 µL of a 10 mM denatonium benzoate solution, prepared in Tyrode buffer (130 mM NaCl, 10 mM HEPES, 10 mM D‐glucose, 5 mM KCl, 1 mM MgCl_2_, 8 mM CaCl_2_, 10 mM sodium pyruvate, 5 mM NaHCO_3_). This solution was directly applied to the tracheal lumen using a pipette. In the in vivo experiments, stimulation was performed using a syringe pump (R‐99, Razel Scientific Instruments, Saint Albans, VT, USA), which deposited ≈20 µL of the denatonium benzoate solution along the length of the trachea.

Imaging time series were captured at a rate of 4.3 fps for in situ experiments, with each frame exposed for 200 ms. For in vivo recordings, to accommodate the increased movement of the animal, time series were recorded at 7.6 fps, with a frame exposure of 100 ms. The 475/28 nm excitation light source was used in combination with a 511/20 nm emission filter, with the LED power set to 47 ± 3 µW at the imaging plane.

### Tuft Cell Distribution

To elucidate the distribution of tuft cells within the mouse trachea, confocal imaging (Figure S2, Supporting Information) was employed on whole‐mount tracheal samples from mice expressing choline acetyltransferase (ChAT)(BAC)‐eGFP in tracheal tuft cells (Jackson strain 0 07902).^[^ ^63^ ^]^

The preparation of whole‐mount tracheal samples for confocal imaging followed the protocol outlined by Hollenhorst et al.^[^ ^31^ ^]^ Tuft cells were visualized by excising the entire trachea from animals with ChAT‐eGFP‐positive tuft cells, then sectioning the trachea into three segments along the cartilage rings. These samples were imaged confocally using a 485 nm laser and a 525/50 nm emission filter on the confocal/STED microscope that was described previously.^[^ ^64^ ^]^

### Neuronal Cell Culture

To evaluate the microendoscope's multichannel capabilities in cell cultures, employing a static fluorophore (mKate) for motion‐artifact detection alongside a calcium sensor (GCaMP6f), primary neuronal cell cultures were prepared as previously described.^[^ ^65^ ^]^ Briefly, hippocampi were extracted from the brains of newborn mouse pups (P0) and digested for 20 min at 37 °C with 10 units of papain (Worthington Biochemical, Lakewood, NJ, USA), followed by gentle mechanical dissociation. The neurons were then seeded at a density of 300 cells mm^−2^ onto 25 mm cover slips coated with 0.5 mg mL^−1^ poly‐D‐lysine (Sigma‐Aldrich). The cultures were incubated at 37 °C in a humidified atmosphere containing 95% air and 5% CO_2_ in NBA medium (Invitrogen, Thermo Fisher Scientific, Waltham, MA, USA), supplemented with 2% B‐27 (Sigma‐Aldrich), 1% GlutaMAX (Invitrogen), 100 U mL^−1^ penicillin and 100 µg mL^−1^ streptomycin (Invitrogen). Neurons were transfected with adeno‐associated virus on the fifth day in culture and imaged between the 12th and 16th day in culture. Cells prepared for calcium imaging with a chemical sensor (Figure S3, Supporting Information) were not incubated with adeno‐associated virus but instead incubated with 1.33 µM Fluo‐4 AM (Ref. F14201, Invitrogen, Thermo Fisher Scientific) in 1 mL Dulbecco's Modified Eagle Medium (Ref. 11 965 092, Gibco, Thermo Fisher Scientific) at 37 °C / 5 % CO_2_ for 30 min.

For imaging, neurons previously transfected with adeno‐associated virus were submerged in an extracellular solution (ECS) composed of 130 mM NaCl, 10 mM NaHCO_3_, 2.4 mM KCl, 2 mM CaCl_2_, 2 mM MgCl_2_, 10 mM HEPES, and 10 mM D‐glucose, with the pH adjusted to 7.3 using NaOH. To induce membrane depolarization, the culture was perfused with a high‐KCl ECS (90 mM NaCl, 10 mM NaHCO_3_, 80 mM KCl, 2 mM CaCl_2_, 2 mM MgCl_2_, 10 mM HEPES, 10 mM D‐glucose, pH adjusted to 7.3 with NaOH). Cells prepared for calcium imaging with a chemical sensor were submerged in ECS without CaCl_2_ to induce spontaneous activity.

The neuronal cultures expressing GCaMP6f and mKate2 (Figure 6a) were imaged using a quad‐band filter set (435/15, 520/10, 595/15, and 695/30), which allowed for simultaneous acquisition of signals from both fluorophores. Illumination alternated between 475/28 nm for GCaMP6f (odd frames) and 555/28 nm for mKate2 (even frames), with an exposure time of 200 ms for each wavelength. This frame‐interleaved two‐channel time series achieved a raw frame rate of 4.3 fps, translating to an effective frame rate of 2.15 fps for each channel. The illumination power of the 475/28 nm LED for GCaMP excitation was set to 47 ± 3 µW at the imaging plane, and the power of the 555/28 nm LED for mKate2 illumination was set to 54 ± 1.9 µW. Neuronal cultures stained with Fluo‐4 AM were imaged using the 511/20 single‐band filter with 475/28 nm LED excitation, at a frame rate of 9.5 frames per second at 75 ms exposure time and 270 ± 1.5 µW in the imaging plane.

The optical characterization of the microendoscope for single‐cell resolution was conducted using a neuronal cell culture preparation similar to that described above, but with the “TRPC5‐IC/eR26‐**τ** GFP line”, as detailed before.^[^ ^65^ ^]^

### Ratiometric Redox Imaging in Whole Organs

Ratiometric imaging was validated with transgenic mice expressing a genetically encoded ratiometric H_2_O_2_ sensor, mito‐roGFP2‐Orp1, localized to the mitochondria.^[^ ^37^ ^]^

Ratiometric redox signals were recorded from various excised organs prepared as follows: Animals were euthanized by cervical dislocation. The pancreas and kidneys were excised after opening the abdominal cavity and dissecting the organs with surgical scissors. The abdomen was shaved, and the skin was carefully held with blunt forceps. Surgical scissors were then used to make horizontal and vertical incisions to expose the abdominal muscles, which were subsequently cut. With the abdominal cavity open, the target organ was identified, and surrounding connective tissue and fat were removed with blunt forceps. The organ was isolated until the only points of attachment were the major blood vessels, which were severed in a single motion to expediently excise the organ. Throughout the procedure, the organs were kept moist with Krebs‐Henseleit buffer (KHB), which consisted of 120 mM NaCl, 4.8 mM KCl, 1.2 mM MgCl_2_, 2.5 mM CaCl_2_, 24 mM NaHCO_3_, 5 mM HEPES, 10 mM glucose, and 1 g L^−1^ bovine serum albumin, with the pH adjusted to 7.35–7.40 using NaOH.

For ratiometric imaging, excised organs and islets were immersed in KHB. To induce oxidative conditions, tetramethylazodicarboxamide (“diamide,” Sigma‐Aldrich, D3648) was added to the KHB to a final concentration of 2 mM. After a buffer exchange, the organs were stimulated with DTT (Sigma‐Aldrich, D9779), a reductive agent, at a final concentration of 10 mM. For diamide stimulation, 50 µL of a 200 mM diamide solution were added to 5 ml of KHB. For subsequent DTT stimulation, the buffer was refreshed, and 100 µL of a 500 mM DTT solution was introduced into 5 mL of KHB.

Ratiometric imaging of mito‐roGFP2‐Orp1 (Figure 6b–d) employed a single‐band filter. Imaging alternated between a 395/35 nm light source for odd frames and a 475/28 nm light source for even frames, each with an exposure time of 200 ms. The illumination intensity was 47 ± 3 µW at the imaging plane with the 475/28 nm LED and 14 ± 1 µW with the 395/25 nm LED.

Images were captured at a rate of 1.8 fps for each channel. This reduced frame rate, compared to those of single‐channel recordings, was slightly more pronounced than the expected factor of two reduction due to the additional processing time required for the live display to visually monitor both channels simultaneously.

### Ratiometric Redox Imaging in Pancreatic Islet Culture

The ratiometric imaging system was also applied to pancreatic islet cultures, where short diffusion times can be easily achieved. To prepare the cultures, the abdomen of the mouse was fully opened following cervical dislocation. The ampulla was pinched off and a small cut was made in the common bile duct to allow for the infusion of a collagenase solution (0.63 mg mL^−1^ collagenase P [Ref: 11 213 865 001], Roche via Sigma, Mannheim, Germany) diluted in KHB,^[^ ^66^ ^]^ supplemented with 100 U mL^−1^ penicillin and 100 µg mL^−1^ streptomycin. This step inflated the pancreas, which was then excised and further digested with collagenase for 20 min at 37 °C in a water bath to maintain a constant temperature. After mechanical homogenization through shaking, the tissue was washed three times with KHB. Pancreatic islets were manually separated from the exocrine tissue and subsequently cultured in RPMI 1640 (Ref: 21 875 034, Gibco, Thermo Fisher Scientific) enriched with 10% fetal bovine serum (Ref: 10 270 106, Gibco, Thermo Fisher Scientific), 100 U mL^−1^ penicillin and 100 µg mL^−1^ streptomycin (P4333; Sigma‐Aldrich) at 37 °C and 5% CO_2_ overnight prior to imaging.

Post‐incubation, groups of ten islets were arranged on a poly‐L‐lysine (Sigma, P4707)‐coated petri dish filled with KHB. The microendoscope, positioned directly above an individual islet, recorded data before and after the introduction of diamide (to a final concentration of 2 mM) and DTT (to a final concentration of 10 mM) into the culture medium, capturing the responses of the islets. Imaging parameters were the same as those used for the whole pancreas.

### Ethical Statement

It is confirmed that all data generated and analyzed in this work adheres to the ethical standards of the Best Practice Guidelines on Research Integrity and Publishing Ethics detailed by Wiley.

## Conflict of Interest

The authors declare no conflict of interest.

## Author Contributions

T.A.D. and M.A.L. conceived and built the microendoscope. T.A.D. performed the in situ experiments and dual‐channel recordings. T.A.D. and M.I.E. performed the in vivo experiments. M.I.E. and G.K.C. provided the tuft‐cell specific mice and established the trachea‐related experiments. R.R. and P.L. provided podocyte specific mice and established the kidney‐related experiments. Y.S. and D.B. prepared neuronal cultures and performed viral infections. M.D.A.H., C.C., and L.P.R. prepared pancreatic islet cultures and assisted pancreas experiments. D.S. provided tracheal whole mount samples for the investigation of tuft cell distribution and assisted the imaging. A.W., V.W., and U.B. tailor‐made AAVs. T.A.D. and M.A.L. wrote the analysis software. Q.T. aided in conceptualizing the microendoscope and wrote the software controlling the Arduino. A.K. helped build the microendoscope and performed the first tests. M.A.L., G.K.C., P.L., M.I.E., and Q.T. conceived the project and wrote the grant proposal. T.A.D. and M.A.L. wrote the manuscript with input from all authors. T.A.D. prepared the figures.

## Supporting information



Supporting Information

Supplemental Video 1

Supplemental Video 2

## Data Availability

The data and code generated for this study are available from the corresponding author upon reasonable request.

## References

[1] V. Subramanian , K. Ragunath , Clin. Gastroenterol. Hepatol. 2014, 12, 368. 23811245 10.1016/j.cgh.2013.06.015

[2] J. Qi , T. Tatla , E. Nissanka‐Jayasuriya , A. Y. Yuan , D. Stoyanov , D. S. Elson , Nat. Biomed. Eng. 2023, 7, 971. 37012312 10.1038/s41551-023-01018-0 PMC10427430

[3] G. Oh , E. Chung , S. H. Yun , Optical Fiber Technology 2013, 19, 760.

[4] Y. Choi , C. Yoon , M. Kim , T. D. Yang , C. Fang‐Yen , R. R. Dasari , K. J. Lee , W. Choi , Phys. Rev. Lett. 2012, 109, 203901. 23215488 10.1103/PhysRevLett.109.203901 PMC4001713

[5] H. Zhang , K. Vyas , G. Z. Yang , J. Biomed. Opt. 2019, 24, 116501. 31724344 10.1117/1.JBO.24.11.116501 PMC7003141

[6] K. Park , J. H. Kim , T. Kong , W. Sun , J. Lee , T. D. Yang , Y. Choi , Biomed. Opt. Express 2020, 11, 4976. 33014594 10.1364/BOE.399428 PMC7510851

[7] A. Shahmoon , S. Aharon , O. Kruchik , M. Hohmann , H. Slovin , A. Douplik , Z. Zalevsky , Sci. Rep. 2013, 3, 1805. 23712369 10.1038/srep01805 PMC3664902

[8] R. S. Pillai , D. Lorenser , D. D. Sampson , Opt. Express 2011, 19, 7213. 21503033 10.1364/OE.19.007213

[9] D. Yelin , I. Rizvi , W. M. White , J. T. Motz , T. Hasan , B. E. Bouma , G. J. Tearney , Nature 2006, 443, 765. 17051200 10.1038/443765a

[10] Y. Li , Y. Yang , W. Li , C. Chen , Q. Lin , H. Huang , Y. Gu , X. Jin , Z. Qian , Biomed. Opt. Express 2024, 15, 3770. 38867773 10.1364/BOE.523179 PMC11166437

[11] N. Krstajic , B. Mills , I. Murray , A. Marshall , D. Norberg , T. H. Craven , P. Emanuel , T. R. Choudhary , G. O. S. Williams , E. Scholefield , A. R. Akram , A. Davie , N. Hirani , A. Bruce , A. Moore , M. Bradley , K. Dhaliwal , J. Biomed. Opt. 2018, 23, 076005. 10.1117/1.JBO.23.7.076005 29992799

[12] J. Li , S. Thiele , R. W. Kirk , B. C. Quirk , A. Hoogendoorn , Y. C. Chen , K. Peter , S. J. Nicholls , J. W. Verjans , P. J. Psaltis , C. Bursill , A. M. Herkommer , H. Giessen , R. A. McLaughlin , Small 2022, 18, 2107032. 10.1002/smll.202107032 35229467

[13] M. K. Quinn , T. C. Bubi , M. C. Pierce , M. K. Kayembe , D. Ramogola‐Masire , R. Richards‐Kortum , PLoS One 2012, 7, 44924. 10.1371/journal.pone.0044924 PMC3445555 23028683

[14] N. Bodenschatz , C. F. Poh , S. Lam , P. Lane , M. Guillaud , C. E. MacAulay , J. Biomed. Opt. 2017, 22, 086005. 10.1117/1.JBO.22.8.086005 28823113

[15] C. Ba , M. Palmiere , J. Ritt , J. Mertz , Biomed. Opt. Express 2016, 7, 3403. 27699107 10.1364/BOE.7.003403 PMC5030019

[16] H. Bao , J. Allen , R. Pattie , R. Vance , M. Gu , Opt. Lett. 2008, 33, 1333. 18552949 10.1364/ol.33.001333

[17] T. M. Urner , A. Inman , B. Lapid , S. Jia , Biomed. Opt. Express 2022, 13, 590. 35284166 10.1364/BOE.447578 PMC8884202

[18] J. Bahlmann , N. Madrahimov , F. Daniel , D. Theidel , D. E. DeTemple , M. Buettner , A. Bleich , A. Haverich , A. Heisterkamp , S. Kalies , Sci. Rep. 2020, 10, 9224. 32513950 10.1038/s41598-020-65950-w PMC7280182

[19] M. Stibůrek , P. Ondrackova , T. Tuckova , S. Turtaev , M. Siler , T. Pikalek , P. Jakl , A. Gomes , J. Krejci , P. Kolbabkova , H. Uhlirova , T. Cizmar , Nat. Commun. 2023, 14, 1897. 37019883 10.1038/s41467-023-36889-z PMC10076269

[20] Z. Wen , Z. Dong , Q. Deng , C. Pang , C. F. Kaminski , X. Xu , H. Yan , L. Wang , S. Liu , J. Tang , W. Chen , X. Liu , Q. Yang , Nat. Photonics 2023, 17, 679.

[21] P. Garg , Am J Nephrol 2018, 47, 3.29852492

[22] V. D. D'Agati , A. Chagnac , A. P. de Vries , M. Levi , E. Porrini , M. Herman‐Edelstein , M. Praga , Nat. Rev. Nephrol. 2016, 12, 453. 27263398 10.1038/nrneph.2016.75

[23] J. Wiggins , Semin. Nephrol. 2009, 29, 587. 20006790 10.1016/j.semnephrol.2009.07.012 PMC2796245

[24] D. V. Ilatovskaya , O. Palygin , V. Levchenko , A. Staruschenko , J Vis Exp 2015, 52850. 26167808 10.3791/52850 PMC4544950

[25] J. Binz‐Lotter , C. Jungst , M. M. Rinschen , S. Koehler , P. Zentis , A. Schauss , B. Schermer , T. Benzing , M. J. Hackl , J. Am. Soc. Nephrol. 2020, 31, 532. 31924670 10.1681/ASN.2019020109 PMC7062224

[26] J. S. Kang , S. J. Lee , J. H. Lee , J. H. Kim , S. S. Son , S. K. Cha , E. S. Lee , C. H. Chung , E. Y. Lee , Sci. Rep. 2019, 9, 7679. 31118506 10.1038/s41598-019-44194-3 PMC6531474

[27] S. Kaske , G. Krasteva , P. Konig , W. Kummer , T. Hofmann , T. Gudermann , V. Chubanov , BMC Neurosci 2007, 8, 49. 17610722 10.1186/1471-2202-8-49 PMC1931605

[28] C. Lasconi , S. Pifferi , A. Hernandez‐Clavijo , F. Merigo , M. P. Cecchini , K. Y. Gonzalez‐Velandia , E. Agostinelli , A. Sbarbati , A. Menini , Sci. Rep. 2019, 9, 8834. 31222082 10.1038/s41598-019-45456-w PMC6586933

[29] M. Deprez , L. E. Zaragosi , M. Truchi , C. Becavin , S. Ruiz Garcia , M. J. Arguel , M. Plaisant , V. Magnone , K. Lebrigand , S. Abelanet , F. Brau , A. Paquet , D. Pe'er , C. H. Marquette , S. Leroy , P. Barbry , Am J. Respir. Crit. Care Med. 2020, 202, 1636. 32726565 10.1164/rccm.201911-2199OC

[30] G. Krasteva , B. J. Canning , P. Hartmann , T. Z. Veres , T. Papadakis , C. Muhlfeld , K. Schliecker , Y. N. Tallini , A. Braun , H. Hackstein , N. Baal , E. Weihe , B. Schutz , M. Kotlikoff , I. Ibanez‐Tallon , W. Kummer , Proc Natl Acad Sci U S A 2011 108, 9478. 10.1073/pnas.1019418108 PMC3111311 21606356

[31] M. I. Hollenhorst , I. Jurastow , R. Nandigama , S. Appenzeller , L. Li , J. Vogel , S. Wiederhold , M. Althaus , M. Empting , J. Altmüller , A. K. H. Hirsch , V. Flockerzi , B. J. Canning , A. E. Saliba , G. Krasteva‐Christ , FASEB J. 2020, 34, 316. 31914675 10.1096/fj.201901314RR

[32] M. I. Hollenhorst , R. Nandigama , S. B. Evers , I. Gamayun , N. Abdel Wadood , A. Salah , M. Pieper , A. Wyatt , A. Stukalov , A. Gebhardt , W. Nadolni , W. Burow , C. Herr , C. Beisswenger , S. Kusumakshi , F. Ectors , T. I. Kichko , L. Hubner , P. Reeh , A. Munder , S. M. Wienhold , M. Witzenrath , R. Bals , V. Flockerzi , T. Gudermann , M. Bischoff , P. Lipp , S. Zierler , V. Chubanov , A. Pichlmair , et al., J. Clin. Invest. 2022, 132, 150951. 10.1172/JCI150951 PMC9246383 35503420

[33] G. T. Hanson , R. Aggeler , D. Oglesbee , M. Cannon , R. A. Capaldi , R. Y. Tsien , S. J. Remington , J. Biol. Chem. 2004, 279, 13044. 14722062 10.1074/jbc.M312846200

[34] K. C. Wagener , B. Kolbrink , K. Dietrich , K. M. Kizina , L. S. Terwitte , B. Kempkes , G. Bao , M. Müller , Antioxid Redox Signal 2016, 25, 41. 27059697 10.1089/ars.2015.6587 PMC4931743

[35] J. P. Deglasse , L. P. Roma , D. Pastor‐Flores , P. Gilon , T. P. Dick , J. C. Jonas , Antioxid Redox Signal 2019, 30, 297. 29756464 10.1089/ars.2017.7287

[36] G. B. Waypa , J. D. Marks , R. Guzy , P. T. Mungai , J. Schriewer , D. Dokic , P. T. Schumacker , Circ. Res. 2010, 106, 526. 20019331 10.1161/CIRCRESAHA.109.206334 PMC2856085

[37] Y. Fujikawa , L. P. Roma , M. C. Sobotta , A. J. Rose , M. B. Diaz , G. Locatelli , M. O. Breckwoldt , T. Misgeld , M. Kerschensteiner , S. Herzig , K. Müller‐Decker , T. P. Dick , Sci Signal 2016, 9, rs1. 26980443 10.1126/scisignal.aad3895

[38] J. A. Udovich , N. D. Kirkpatrick , A. Kano , A. Tanbakuchi , U. Utzinger , A. F. Gmitro , Appl. Opt. 2008, 47, 4560. 18758526 10.1364/ao.47.004560

[39] K. Matsushita , K. Golgotiu , D. J. Orton , R. D. Smith , K. D. Rodland , P. D. Piehowski , M. P. Hutchens , J Vis Exp 2018, 58206. 30371667 10.3791/58206 PMC6235460

[40] F. Wang , R. Jiang , K. Takahashi , J. Gore , R. C. Harris , T. Takahashi , C. C. Quarles , Magn Reson Imaging 2014, 32, 1125. 25093632 10.1016/j.mri.2014.07.012 PMC4171209

[41] X. Liang , H. Wang , Y. Zhu , R. Zhang , V. C. Cogger , X. Liu , Z. P. Xu , J. E. Grice , M. S. Roberts , ACS Nano 2016, 10, 387. 26743581 10.1021/acsnano.5b05066

[42] C. N. Liu , J. Morin , M. Dokmanovich , C. T. Bluette , R. Goldstein , B. Manickam , C. M. Bagi , J. Pharmacol. Toxicol. Methods 2019, 96, 67. 30738209 10.1016/j.vascn.2019.02.003

[43] S. J. Schunk , S. Triem , D. Schmit , S. Zewinger , T. Sarakpi , E. Becker , G. Hutter , S. Wrublewsky , F. Küting , M. Hohl , D. Alansary , L. Prates Roma , P. Lipp , J. Möllmann , M. Lehrke , M. W. Laschke , M. D. Menger , R. Kramann , P. Boor , W. Jahnen‐Dechent , W. März , M. Bohm , U. Laufs , B. A. Niemeyer , D. Fliser , E. Ampofo , T. Speer , Circulation 2021, 144, 893. 34192892 10.1161/CIRCULATIONAHA.121.053547

[44] D. V. Ilatovskaya , O. Palygin , V. Levchenko , B. T. Endres , A. Staruschenko , Sci. Rep. 2017, 7, 299. 28331185 10.1038/s41598-017-00406-2 PMC5428415

[45] J. Binz‐Lotter , C. Jüngst , M. M. Rinschen , S. Koehler , P. Zentis , A. Schauss , B. Schermer , T. Benzing , M. J. Hackl , J. Am. Soc. Nephrol. 2020, 31, 532. 31924670 10.1681/ASN.2019020109 PMC7062224

[46] W. Kuo , N. A. Le , B. Spingler , R. H. Wenger , A. Kipar , U. Hetzel , G. Schulz , B. Müller , V. Kurtcuoglu , Microsc. Microanal. 2020, 26, 731. 32627730 10.1017/S1431927620001725

[47] H. Bao , J. Allen , R. Pattie , R. Vance , M. Gu , Opt. Lett. 2008, 33, 1333. 18552949 10.1364/ol.33.001333

[48] A. Thrapp , M. Hughes , J. Biomed. Opt. 2021, 26, 056501. 33988004 10.1117/1.JBO.26.5.056501 PMC8116667

[49] P. M. Lane , A. L. P. Dlugan , R. Richards‐Kortum , C. E. MacAulay , Opt. Lett. 2000, 25, 1780. 18066342 10.1364/ol.25.001780

[50] S. Lee , G. Courties , M. Nahrendorf , R. Weissleder , C. Vinegoni , J. Biomed. Opt. 2017, 22, 036005. 28253383 10.1117/1.JBO.22.3.036005 PMC5333764

[51] D. Aharoni , T. M. Hoogland , Front Cell Neurosci 2019, 13, 141. 31024265 10.3389/fncel.2019.00141 PMC6461004

[52] S. Cherepanov , L. Heitzmann , P. Fontanaud , A. Guillou , E. Galibert , P. Campos , P. Mollard , A. O. Martin , iScience 2024, 27, 109876. 38799572 10.1016/j.isci.2024.109876 PMC11126972

[53] G. Liu , S. W. Ahn , J. W. Kang , S. Bhagavatula , D. Matthew , S. Martin , C. Marlin , P. T. C. So , G. J. Tearney , O. Jonas , Opt. Lett. 2024, 49, 3312. 38875608 10.1364/OL.525945 PMC11298057

[54] T. Lambert , mKate2 at FPbase, https://www.fpbase.org/protein/mkate2/ (accessed: October 2023).

[55] T. Lambert , EGFP at FPbase. https://www.fpbase.org/protein/egfp/(accessed: October 2023).

[56] M. J. Moeller , S. K. Sanden , A. Soofi , R. C. Wiggins , L. B. Holzman , Genesis 2003, 35, 39. 12481297 10.1002/gene.10164

[57] M. Paukert , A. Agarwal , J. Cha , V. A. Doze , J. U. Kang , D. E. Bergles , Neuron 2014, 82, 1263. 24945771 10.1016/j.neuron.2014.04.038 PMC4080721

[58] J. Czogalla , F. Schweda , J. Loffing , J Vis Exp 2016, 54712. 27911373 10.3791/54712 PMC5226255

[59] R. Plenter , S. Jain , C. M. Ruller , T. L. Nydam , A. H. Jani , J Vis Exp 2015, 52848. 26555373 10.3791/52848 PMC4692659

[60] S. Kusumakshi , A. Voigt , S. Hubner , I. Hermans‐Borgmeyer , A. Ortalli , M. Pyrski , J. Dorr , F. Zufall , V. Flockerzi , W. Meyerhof , J. P. Montmayeur , U. Boehm , Chem Senses 2015, 40, 413. 25940069 10.1093/chemse/bjv023

[61] E. W. Hughes , R. L. Martin‐Body , I. H. Sarelius , J. D. Sinclair , Clin. Exp. Pharmacol. Physiol. 1982, 9, 119. 6813006 10.1111/j.1440-1681.1982.tb00788.x

[62] H. N. Sapru , A. J. Krieger , Eur. J. Pharmacol. 1979, 53, 151. 759195 10.1016/0014-2999(79)90160-2

[63] Y. N. Tallini , B. Shui , K. S. Greene , K.‐Y. Deng , R. Doran , P. J. Fisher , W. Zipfel , M. I. Kotlikoff , Physiological Genomics 2006, 27, 391. 16940431 10.1152/physiolgenomics.00092.2006

[64] A. Staudt , O. Ratai , A. Bouzouina , C. Fecher‐Trost , A. Shaaban , H. Bzeih , A. Horn , A. H. Shaib , M. Klose , V. Flockerzi , M. A. Lauterbach , J. Rettig , U. Becherer , Front Mol Neurosci 2022, 15, 674243. 35493323 10.3389/fnmol.2022.674243 PMC9049930

[65] Y. Schwarz , K. Oleinikov , B. Schindeldecker , A. Wyatt , P. Weissgerber , V. Flockerzi , U. Boehm , M. Freichel , D. Bruns , PLoS Biol. 2019, 17, 3000445. 10.1371/journal.pbio.3000445 PMC6773422 31536487

[66] L. E. Bailey , S. D. Ong , J. Pharmacol. Methods 1978, 1, 171.

