# Supplementary material for: Functional Microendoscopy Reveals Calcium Responses of Single Cells in Tracheal Tuft Cells and Kidney Podocytes
Source: Small. 2025 Apr 1;21(21):2411341. doi: 10.1002/smll.202411341 (PMC12105425; doi:10.1002/smll.202411341)
Supplement: Supplementary file 1 — Supporting Information [file SMLL-21-2411341-s002.pdf]

NANO · MICRO
small

Supporting Information

for *Small*, DOI 10.1002/smll.202411341

Functional Microendoscopy Reveals Calcium Responses of Single Cells in Tracheal Tuft Cells and Kidney Podocytes

*Tobias A. Dancker, Mohamed Ibrahem Elhawy, Ramona Rittershauß, Qinghai Tian, Yvonne Schwarz, Markus D. A. Hoffmann, Christopher Carlein, Amanda Wyatt, Vanessa Wahl, Daniel Speyerer, Alaa Kandah, Ulrich Boehm, Leticia Prates Roma, Dieter Bruns, Peter Lipp, Gabriela Krasteva-Christ and Marcel A. Lauterbach**

Supporting Information

Functional Microendoscopy Reveals Calcium Responses of Single Cells in Tracheal Tuft Cells and Kidney Podocytes

*Tobias A. Dancker, Mohamed Ibrahem Elhawy, Ramona Rittershauß, Qinghai Tian, Yvonne Schwarz, Markus D. A. Hoffmann, Christopher Carlein, Amanda Wyatt, Vanessa Wahl, Daniel Speyerer, Alaa Kandah, Ulrich Boehm, Leticia Prates Roma, Dieter Bruns, Peter Lipp, Gabriela Krasteva-Christ, Marcel A. Lauterbach**

Contents

Figure S1: Overlay of spectra

Figure S2: Whole-mount trachea for density estimation of tuft cells

Figure S3: Fluo-4 AM neuronal calcium signal

Figure S4: Raw traces of calcium signals

Video S1: In situ tuft cell response to denatonium stimulation

Video S2: In vivo tuft cell response to denatonium stimulation

Table S1: Summary of fluorophores and filters

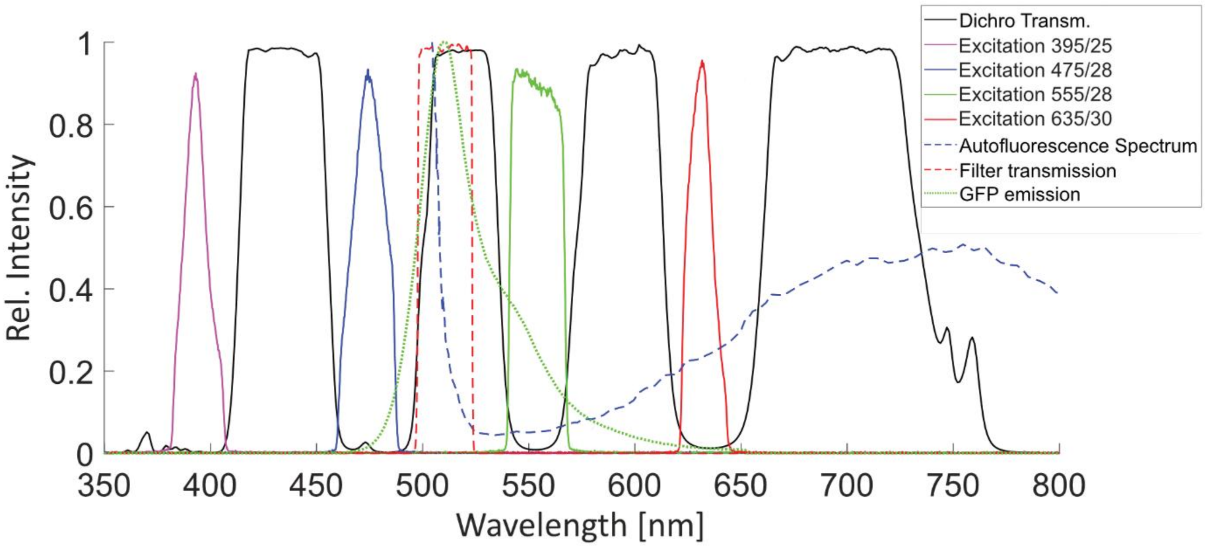

Figure S1. Emission filter transmission overlaid with dichroic mirror transmission, fiber autofluorescence spectrum, excitation wavelengths, and emission spectrum of GFP.

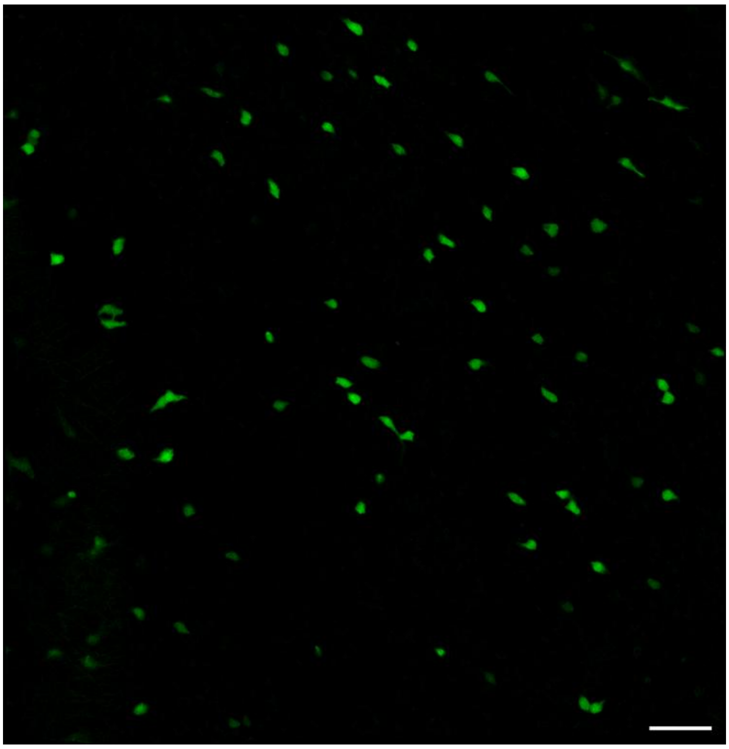

Figure S2. Confocal image of tuft cells in a fixed whole-mount mouse trachea, revealing a sparse distribution. Scale bar: 50 µm.

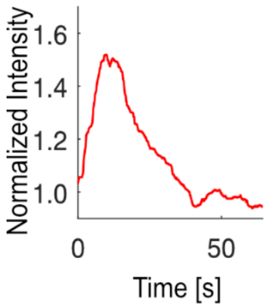

Figure S3. Neuronal calcium signal (spontaneous activity of an individual neuron) recorded with the chemical sensor Fluo-4 AM.

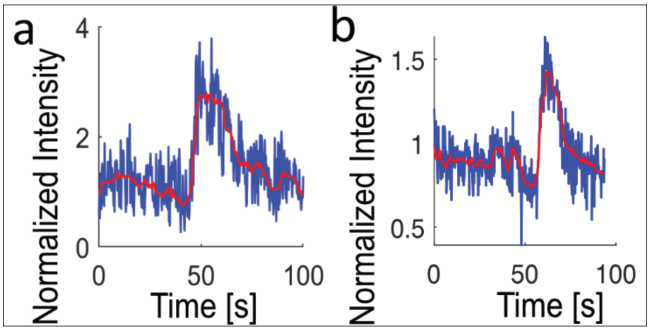

Figure S4. (a) Single podocyte response to stimulation with angiotensin II. The blue trace shows the raw recorded calcium signal, while the red trace shows the filtered signal [cf. Figure 3 (d)]. (b) Calcium response of a single tracheal tuft cell upon stimulation with denatonium. The blue trace shows the raw recorded calcium signal, while the red trace shows the filtered signal [cf. Figure 4 (e)].

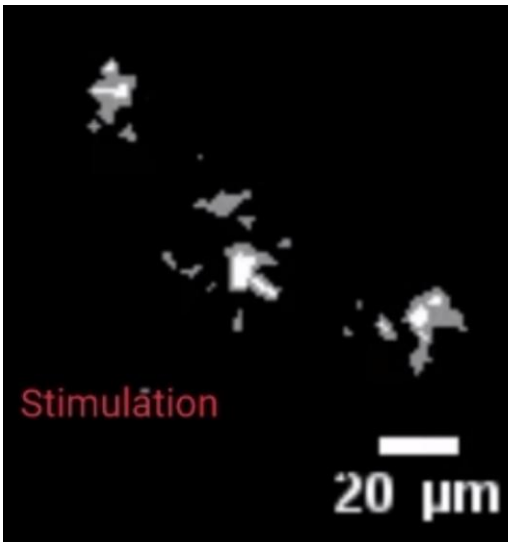

Video S1. Microendoscopic recording of in situ tuft cell stimulation. The trachea is stimulated with a 4 µl droplet of 10 mM denatonium benzoate in situ. Scale bar: 20 µm.

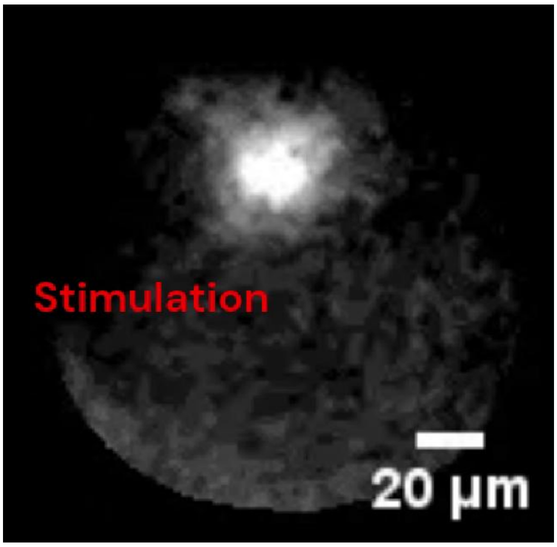

Video S2. Microendoscopic recording of in vivo tuft cell stimulation. The trachea is stimulated with a 2 µl/min flow of denatonium benzoate in vivo. Scale bar: 20 µm.

Table S1: Fluorophores used, the function they served, and the excitation and emission filters used for each fluorophore

Fluorophores	Function in experiment	Excitation filter [nm]	Emission filter	Corresponding figure
GCaMP3/ GCaMP6f	Deliver information about cellular calcium signaling	475/28	511/20 single-band filter (F39-509, AHF analysentechnik) 435/15, 520/10, 595/15, and 695/30 quad-band filter (F67-401, AHF analysentechnik)	Figures 3, 4, 5 and 6, Supplementary Figure 4
Fluo-4 AM	Deliver information about cellular calcium signaling	475/28	511/20 single-band filter (F39-509, AHF analysentechnik)	Supplementary Figure 3
roGFP2-Orp1	Detect ratiometrically cellular H_2O_2 redox variations	395/25 and 475/28	511/20 single-band filter (F39-509, AHF analysentechnik)	Figure 6
mKate2	Provide a static reference to a dynamic signal to exclude movement artifacts.	555/28	435/15, 520/10, 595/15, and 695/30 quad-band filter (F67-401, AHF analysentechnik)	Figure 6
Yellow green (505/515) FluoSpheres	Provide a fluorescent point source to assess microendoscope resolution	475/28	511/20 single-band filter (F39-509, AHF analysentechnik)	Figure 2
